# The effect of an acute aspirin challenge on intestinal permeability in healthy adults with and without prophylactic probiotic consumption: a double-blind, placebo-controlled, randomized trial

**DOI:** 10.1186/s12876-023-03102-w

**Published:** 2024-01-02

**Authors:** Taylor C. Judkins, Rebecca J. Solch-Ottaiano, Brendan Ceretto-Clark, Carmelo Nieves, James Colee, Yu Wang, Thomas A. Tompkins, Sara E. Caballero-Calero, Bobbi Langkamp-Henken

**Affiliations:** 1 Food Science and Human Nutrition Department, University of Florida, 572 Newell Dr, Gainesville, FL 32611 USA; 2 IFAS Statistical Consulting Unit, University of Florida, PO Box 110500, Gainesville, FL 32611-0500 USA; 3 Citrus Research and Education Center, Institute of Food and Agricultural Sciences, University of Florida, Lake Alfred, FL 33850 USA; 4 Lallemand Bio-Ingredients, 1620 Rue Prefontaine, Montreal, QC H1N 2W8 Canada; 5 Lallemand Health Solutions, 6100 Royalmount Avenue, Montreal, QC H4P 2R2 Canada

**Keywords:** Intestinal permeability, NSAID, Gastrointestinal health, Probiotic

## Abstract

**Background:**

Healthy individuals may experience increases in intestinal permeability after chronic or acute use of non-steroidal anti-inflammatory drugs, which may be attenuated by probiotics. This study investigates the effects of an acute aspirin challenge on gastroduodenal barrier function with or without prophylactic probiotic consumption.

**Methods:**

Twenty-nine generally healthy participants (26 ± 6 years) completed a 14-week randomized, double-blind, crossover trial. A probiotic containing 2 *Lactobacilli* strains or placebo was administered for 3 weeks, with a 4-week washout period between crossover phases. Daily and weekly questionnaires assessing gastrointestinal function were completed for 2 weeks before until 2 weeks after each intervention to assess gastrointestinal function. Gastroduodenal permeability was assessed by urinary excretion of orally administered sucrose after 1, 2, and 3 weeks of each intervention with a 1950 mg-aspirin challenge after 2 weeks of supplementation. Stool samples were collected weekly during supplementation for detection of species of interest.

**Results:**

Gastroduodenal permeability increased with aspirin challenge (Week 1: 3.4 ± 0.6 μmol vs Week 2: 9.9 ± 1.0 μmol urinary sucrose; *p* < 0.05). There were no differences in the change in permeability after the aspirin challenge or gastrointestinal function between interventions.

**Conclusion:**

The acute aspirin challenge significantly increased intestinal permeability similarly in both groups, and prophylactic probiotic consumption was unable to prevent the loss in this particular model.

**Supplementary Information:**

The online version contains supplementary material available at 10.1186/s12876-023-03102-w.

## Background

The intestinal barrier is a dynamic structure that works to separate the external milieu from the internal environment. In addition to the epithelial barrier regulating entry of water and nutrients, mucus and immunological and microbial components work to prevent entry of pathogenic bacteria and other pro-inflammatory substances [1]. Several chronic immune and metabolic disorders, such as inflammatory bowel disease, type 2 diabetes, and nonalcoholic fatty liver disease have been associated with a disruption of the intestinal barrier resulting in increased intestinal permeability [2–4]. Chronically increased intestinal permeability is generally accepted to negatively impact the host due to its associated increases in systemic inflammation [5]. Individuals who are generally healthy may also have acute increases in intestinal permeability. Activities such as running and exercising, general life stress, and ingestion of medications such as nonsteroidal anti-inflammatory drugs (NSAID) have been shown to increase intestinal permeability [6–8].

NSAID, such as aspirin, ibuprofen, and naproxen sodium, are over the counter pain relievers generally used for headache, body aches, swelling, stiffness and fever. It is estimated that over 6 million people in the United States alone consume aspirin without the recommendation of a physician [9]. The maximum suggested dose of aspirin is 4,000 mg per day for short-term treatment (typically 3–6 days), consumed in 3 to 4 doses over 24 h, and while it is generally viewed as safe, it may cause gastrointestinal (GI) disturbances and increase intestinal permeability [10, 11]. Aspirin is absorbed by the gastroduodenal mucosa, where it can directly injure the epithelium. However, the damage is not limited to the gastroduodenal mucosa as NSAID also induce a loss of small intestinal barrier function [11, 12]. In 2018, Bjarnason and colleagues put forth a model to explain the various mechanisms by which NSAID cause GI damage and an increase in permeability [12]. They hypothesized that NSAID interact with the phospholipids in the mucus and membrane layers and uncouple mitochondrial oxidative phosphorylation resulting in damage to cells and increased GI permeability [12]. Further, the inhibition of cyclooxygenase, the rate-limiting enzyme in the formation of prostaglandins, reduces microvascular blood flow. The damage to the mucosa and reduction in blood flow allow acid and pepsin in the stomach and acid, bile, and bacteria in the small intestine to intensify the damage [12]. An additional mechanism for loss of barrier function may be from the effect of aspirin on tight junction proteins. In human gastric epithelial cell lines, aspirin increased dextran permeability in a dose-dependent manner and decreased the tight junction protein claudin-7. Pretreatment with a p38 MAPK-specific inhibitor abolished these effects [13].

NSAID may also indirectly damage the intestinal epithelium by altering microbial composition and giving rise to gram-negative bacterial overgrowth. This may result in an increase in endotoxin which triggers the recruitment of neutrophils and the release of proteases and reactive oxygen species that ultimately injure the intestinal epithelium [14–18]. The microbial composition of the gut is therefore influential on the gut barrier suggesting that probiotics may be useful to protect against perturbations that increase intestinal permeability [19]. Probiotics thrive in large numbers in the colon, however, they also exert numerous beneficial effects within the proximal intestine [20]. Because NSAID damage the proximal small intestine, a probiotic that exhibits effects in the proximal as well as the distal intestine is of interest [12]. *Lactobacillus* a gram-positive species may be good candidates as it persists along the entirety of the GI tract, increases diversity of microbes associated with health, maintains tight junction integrity, and plays a critical role in maintaining whole gut immune function [21–27]. A commercially available probiotic combination containing *Lactobacillus helveticus R0052* and *L. rhamnosus* R0011, was hypothesized to be a potential candidate to maintain intestinal permeability due to its ability to maintain intestinal wall integrity in previous in vitro and in vivo work [28]. In vitro, *L. helveticus* R0052 can adhere to human intestinal cells thereby outcompeting various pathogens and decreasing proinflammatory cytokines while *L. rhamnosus* R0011 maintains intestinal permeability as measured by trans-epithelial electrical resistance and decreases pro-inflammatory cytokines [28–32]. Moreover, the secretome of *L. rhamnosus* R0011 was shown to attenuate the deleterious effect of the *S. typhimurium* secretome on inflammatory markers, trans-epithelial resistance, and tight junction proteins expression in T84 epithelial cells [33]. In Sprague–Dawley rat pups under psychological stress, this probiotic combination attenuated the increase in corticosterone and intestinal permeability [34] and in adult male Brown Norway rats, bacterial adherence to the intestine and subsequent translocation of pathogens to the mesenteric lymph nodes was prevented [35]. In humans with GI diseases, this probiotic combination restored the microbiome resulting in decreased numbers of pathogenic bacteria, such as *Clostridium difficile* and *Heliobacter pylori* [36, 37]. In studies of humans with antibiotic-associated diarrhea or intestinal infection, the probiotic improved GI symptoms and increased anti-inflammatory cytokines and secretory IgA in the stool [38–41].

Considering the demonstrated ability of these probiotic strains to maintain intestinal epithelial integrity and a healthy immune state in various models of stress, we investigated the ability of this formulation to prevent or mitigate the effects of an acute aspirin challenge on intestinal permeability. Specifically, it was hypothesized that prophylactic probiotic consumption before an aspirin challenge would maintain gastroduodenal barrier function.

## Methods

### Study participants

**Fig. 1:**
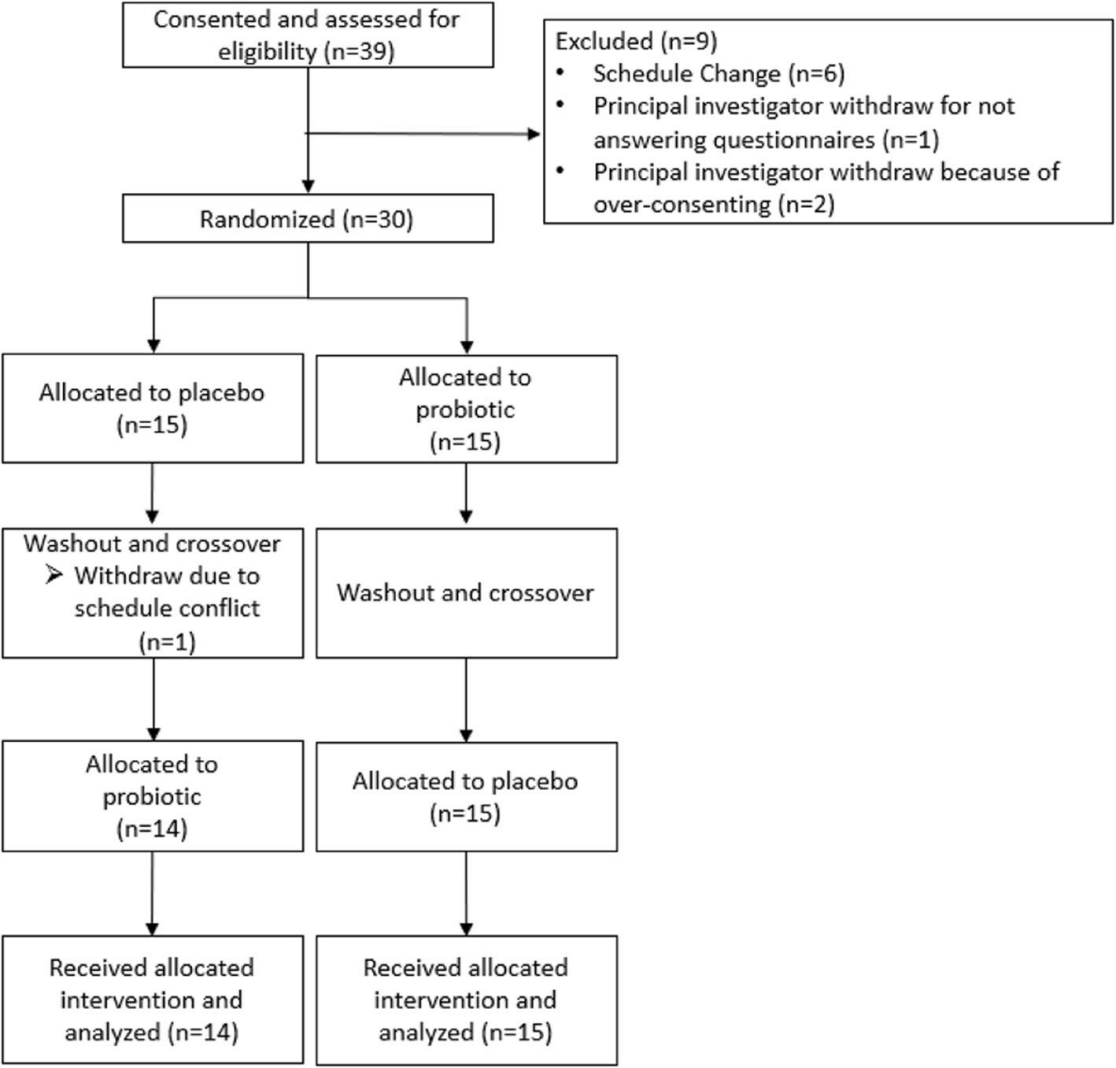
Participant flow diagram. All participants completed a 2-week baseline period. Participants were then randomly allocated to receive the probiotic or placebo supplement and were later allocated to receive the alternative supplement in this crossover study design. The intervention periods (3 weeks each) were separated by a 4-week washout period (2-weeks of washout followed by 2 weeks of a baseline period before the second intervention). The study concluded with a final 2-week washout period. Participants were recruited and completed the study between 2018 and 2019

Participants were recruited (Fig. 1) from a community in the southeastern United States. Written informed consent was obtained by study coordinators and inclusion and exclusion criteria were reviewed for eligibility. Participants qualified for the study if they 1) were 21 to 50 years of age and self-identified as healthy; 2) typically had greater than or equal to 6 bowel movements per week; 3) were willing to discontinue the use of non-study NSAID for the full duration of the study; 4) were willing to avoid the use of antidiarrheal or laxative medications on a regular or as needed basis during the study; 5) were willing to provide urine and stool samples during collection periods; 6) had used aspirin in the past without experiencing adverse events; 7) were willing to consume 1950 mg of aspirin in a 12-h period; 8) were willing to complete online questionnaires each day during the study; 9) were willing to discontinue the consumption of fermented foods, probiotics or probiotic-supplemented foods (live active cultures), prebiotic supplements, herbal supplements, or high-dose vitamin or mineral supplements that may impact immune function or inflammation; 10) were willing to avoid high intensity exercises 2 days prior to each permeability test; 11) were willing to avoid alcohol consumption 2 days prior to each permeability test; 12) were willing to take the study supplement for 6 weeks; and 13) willing to provide informed consent in English. Participants were not eligible to participate if they 1) had a history of or currently had impaired cardiovascular circulation or uncontrolled hypertension, diabetes, bleeding tendencies, kidney, liver or chronic respiratory diseases including asthma, GI disorders including heartburn, or any other disease, that by the investigator’s judgment could interfere with the intestinal barrier function; 2) used NSAID daily in the last 3 months or incidentally consumed an NSAID 2 weeks prior to signing the consent form; 3) consumed medications (not including oral contraceptive pills or a standard multi-vitamin/mineral supplement) 2 weeks prior to the baseline period of the study; 4) had a known sensitivity to gluten or allergy to aspirin, milk, yeast, or soy; 5) were currently smoking; 6) were lactating, known to be pregnant or attempting to become pregnant; or 7) used another investigational product within 3 months of signing the consent form. This study was conducted in accordance with the guidelines included in the Declaration of Helsinki of 1975 as revised in 2013 [42], was approved by the Institutional Review Board at The University of Florida and registered on Clinicaltrials.gov (NCT03611400) on 02/08/2018.

### Study intervention

The study capsules contained either the probiotic or the placebo. Both the capsules and the bottles were identical in weight, shape, color, and presentation. Participants were advised to store the study capsules in the refrigerator for the duration of the study. Each capsule of the probiotic supplement contained a combination of at least 4 billion CFU of *Lacticaseibacillus rhamnosus* R0011 and *Lactobacillus helveticus* R0052. In addition, the probiotic and placebo contained ascorbic acid, hypromellose, magnesium stearate, saccharose, potato starch, titanium dioxide, and maltodextrin as excipients. Both products, which were supplied by Lallemand Health Solutions (Montréal, Canada), may have come into contact with milk, soy, and yeast allergens during the manufacturing process. Participants were instructed to consume one capsule in the morning and one in the evening with a meal. If the participant forgot to take a capsule at any point during the intervention, they were counseled to take it as soon as they remembered.

### Study design and randomization

This study was a 14-week prospective, double-blind, placebo-controlled, crossover study with two 7-week arms that consisted of a 2-week baseline period followed by a 3-week intervention period (2 weeks before the aspirin challenge and 1 week after) and a 2-week washout. This design allowed for a 4-week washout between the first and second intervention. At the initial 2-week baseline period participants began completing online daily and weekly questionnaires and continued to complete these questionnaires for the entire 14-week study. After the initial baseline period, participants were stratified by sex and randomly allocated 1:1 to 1 of 4 number code pairs representing the order of the interventions. A member of the academic institution who was not part of the study team set the randomization scheme using a random-number generator in Excel (Microsoft) and prepared the sealed envelopes. The study team, principal investigator, and sponsor personnel involved in this research protocol remained blinded through the duration of the study and throughout data analysis.

After the baseline periods participants began consuming the assigned study capsules daily for 3 weeks. After each 3-week intervention, participants returned any remaining study capsules. Intestinal permeability was assessed on days 7, 14, and 21 after beginning the study capsules during each intervention period. Day 14 included an aspirin challenge for which all participants were asked to consume 975 mg of non-enterically coated aspirin before bed the night before and then fast overnight or at least 8 h prior to their visit time. Thirty minutes before beginning the day 14 intestinal permeability test and while still fasting, study coordinators had participants consume an additional 975 mg of aspirin.

### Questionnaires

Daily questionnaires recorded the number of study supplements consumed per day, self-reported stress (0 = no stress to 10 = extremely stressed), number of stools, stool form as measured by the Bristol Stool Form Scale (BSFS) (1 = hard stool to 7 = watery stool) [43], and adverse medical events. Weekly questionnaires instructed participants to record information based on the previous week and were comprised of the Gastrointestinal Symptom Rating Scale (GSRS) [44] and the Digestion-Associated Quality of Life Questionnaire (DQLQ) [45]. The GSRS is a 15-item questionnaire that assesses GI symptoms on a scale from 1 (no discomfort at all) to 7 (very severe discomfort) for five syndromes which include abdominal pain, diarrhea, constipation, indigestion, and reflux. The DQLQ is a 9-item questionnaire that assesses how digestive symptoms impacted daily life over the past week based on a scale from never (0% of the time) to always (100% of the time). Percent of time was converted to numbers (0 = never to 1 = always). The total score represents the sum of the 9 questions for a maximum score of 9. Higher scores reflect a lower DQLQ. The International Physical Activity Questionnaire (IPAQ) [46] and a fiber screener [47], which assess physical activity levels and fiber consumption, respectively, over the past week were administered weekly during each intervention. On the final study visits of each intervention period, participants completed the Perceived Stress Scale Questionnaire (PSS) [48] to determine whether stress levels changed over the past month.

Compliance was assessed using participant self-reports in the daily questionnaire and by counting the returned study supplement capsules after each intervention. If a daily questionnaire was not returned, it was assumed that the study supplement was not consumed on that day. In the event of a discrepancy between the information in the daily questionnaire and the number of returned study capsules, compliance was determined based on the capsules returned unless an explanation for the loss of study capsules was provided. Participants were considered compliant if they reported consumption of 90% of the appropriate number of study capsules and completed at least 95% of their daily and weekly questionnaires.

### Permeability testing

An intestinal permeability test was conducted using a sugar cocktail on days 7, 14, and 21 after beginning the study capsules during each intervention period as previously described [49, 50]. In brief, the sugar cocktail consisted of 150 mL of water and food grade sugars. Final weights of sugar probes in the cocktail were adjusted for purity based on information provided by the manufacturer to provide 1 g of sucrose (Now Real Food, IL, USA), lactulose (Wockhardt, IL, USA), sucralose (Micro Ingredients, CA, USA), and erythritol (Now Real Food, IL, USA) and 0.5 g L-rhamnose (Yundao Production, China). The manufacturer- stated purity for sucrose, sucralose, erythritol, lactulose and L-rhamnose was 100%, 100%, 100%, 67%, and 100%, respectively. Each gram of lactulose contained no more than 0.12 g of lactose [51]. At the conclusion of the study, it was determined that the purity of sucrose, sucralose, erythritol and lactulose were > 95%, > 90%, > 95% and 61%, respectively. However, it was also determined at that point that L-rhamnose had been mis-labeled by the manufacturer and contained neotame, a non-nutritive Food and Drug Administration-approved food additive. Participants were notified of this error. Erythritol was used in place of L-rhamnose in accordance with previously described methods [50, 52].

Prior to each permeability test, participants were asked to empty their bladder and then drink the sugar cocktail. Urine was collected for the first 5 h while the participant remained fasted. Separate urine collection jars were used for the first 5 h and the remaining 19 h of the 24-h collection. Urine was kept on ice packs and in coolers until it was dropped off at the study site. The urine volume was measured and 1 mL samples of urine from the 0 to 5-h and 5 to 24-h collection jugs were obtained and stored at -80 °C until analysis. Urine samples were extracted in triplicate and the sugar probes were quantitated using liquid chromatography with tandem mass spectrometry as previously described [50]. Urinary outputs of sucrose and the ratio of lactulose to erythritol in the 0 to 5-h collection were used as an assessment of gastroduodenal and small intestinal permeability, respectively. Urinary outputs of sucralose to erythritol in the 5 to 24-h collection and the entire 24-h collection were used as an assessment of colonic and whole gut permeability, respectively.

### Fecal analyses

#### Microbiome analysis

The first stool sample produced following the ingestion of the sugar cocktail was provided by participants. Stool collection kits (Fisher Scientific) were supplied, and participants kept the samples on ice and delivered them within 4 h of defecation. Samples were homogenized, aliquoted, and stored at -80 °C within 6 h of defecation. Samples were then shipped on dry ice to Lallemand Health Solutions for analyses. DNA was extracted in 5 mL aliquots of thawed stool using the QIAamp® Fast DNA Stool Mini Kit (Qiagen) with two alterations which were washes with 500 mM sodium phosphate buffer prior to the addition of InhibitEX (Qiagen), and a 0.1 mm zirconia/silica bead beating step (~ 300 mg/tube, 4 m/s for 1 min × 3) following incubation with InhibitEx. DNA concentrations were determined using a Nanodrop (Thermo Scientific), and samples were stored at -20 °C. Samples were diluted fivefold in molecular biology grade water prior to qPCR.

Relative quantification was carried out for *Akkermansia muciniphila, Faecalibacterium prausnitzii, Roseburia* spp., and total *Bifidobacterium* spp. DNA was normalized using 16S rDNA Universal Bacterial primers to determine the relative fold change in microbes of interest when comparing each intervention period. Primer sequences and assay conditions for *A. muciniphila* [53],* F. prausnitzii* [54]*,* and *Rosburia* spp. [55] were obtained from previous studies. The *Bifidobacterium* spp. primers were designed by Lallemand Health Solutions (Forward: TGG AAG GTC TCG ATG GAG GT and Reverse: CTG GAC AAG CCG TTC CTG AT). The qPCR reaction mixture (10 μL) consisted of 300 nM of the appropriate primers, 1X SYBR® Select Master Mix (Thermo Fisher Scientific) and 1 μL of diluted DNA. Standard curve samples were analyzed in duplicate and unknown samples, in triplicate. Cycling conditions consisted of initial incubations at 50 °C and 95 °C for 2 min each, followed by 40 cycles of 95 °C for 15 s, 60 °C for 30 s and 72 °C for 30 s. A dissociation curve analysis (60 °C to 95 °C) was also performed at the end of each run to ensure primer specificity All qPCR reactions were prepared using the epMotion 5075tc liquid handing robot (Eppendorf) and SYBR Select® Mastermix, (Thermo Fisher Scientific) and analyzed on the CFX384 Touch ™ Real-Time PCR Detection System (Bio-Rad Laboratories). Fold change in species of interest for the probiotic with respect to the placebo intervention was determined using the Delta Delta Ct method.

### Statistical analysis

#### Sample size

Based on data from a previous study [56], a sample size of 19 would be required to see a significant (*p* ≤ 0.05 and 80% power) difference of 117 μmol of urinary sucrose between the changes (week 2 of intervention with aspirin minus week 1 of intervention without aspirin) with the probiotic versus the placebo interventions. To account for possible attrition due to the rigor of the study design, additional participants were included.

#### Blinding

Upon completion of the study, participants were asked what order they believed they consumed the probiotic and placebo supplement. A Fisher’s Exact Test was performed between the two possible responses.

#### Permeability analyses

The primary outcome, gastroduodenal permeability change, was measured as sucrose output from day 14 (aspirin challenge) of each intervention minus sucrose output from day 7 (no aspirin challenge) of each intervention. A linear mixed model was used. The fixed effects tested in the model were intervention, order, order by intervention, sex, and sex by intervention. For secondary outcomes, fixed effects tested in the model were intervention, order, order by intervention, sex, sex by intervention, week, and week by intervention. For both the primary and secondary outcomes, participants were treated as a random effect to adjust for individual effects between the two intervention periods. All interactions were left in the model regardless of significance.

#### Questionnaire analyses

For all daily and weekly questionnaire outcomes, a linear mixed model was used to test for a week effect between interventions. The fixed effects tested in the model were week, intervention, week by intervention, baseline values, and sex with participant as a random effect. Baseline values included the average value reported during the 2-week baseline periods before each intervention. Baseline values were included in the model to control for potential differences in individuals at baseline.

The PSS was compared between each intervention using a paired t-test. Scores from the fiber screener and the IPAQ were averaged across all 3 weeks during each intervention period and compared between interventions using a paired t-test.

#### Microbiota analysis

Due to the high variability in bacterial species of interest from stool samples collected on days 7, 14, and 21, data from these days were pooled to obtain the mean fold change of microbes of interest across the probiotic intervention with respect to the placebo. If bacteria DNA was undetected in a sample, a value of zero was assumed. A one-sample t-test was used to determine whether the mean fold change was different from 1.

Unless stated otherwise, all data from the permeability, questionnaire, and microbiota analyses are expressed as mean ± SEM. Sigma Plot v12.5 (Systat Software Inc., San Jose, CA, USA) and SAS v9.4 (SAS Institute Inc., Cary, NC, USA) were used for all analyses. Data were analyzed on an intent-to-treat basis and significance of all statistical tests was determined using a type I error rate cut-off of 0.05.

## Results

### Participant characteristics, compliance, and blinding

Thirty-nine participants consented for the study and 30 participants were randomized (Fig. 1). After randomization, 1 participant was withdrawn half-way through this study due to a schedule conflict. Twenty-nine participants completed the study (Table 1). Dietary fiber consumption was not significantly different between the intervention periods (*p* = 0.50, Table 2). Similarly, physical activity, measured by MET-minutes, was not significantly different between the intervention periods (*p* = 0.29, Table 2). Based on all the participants, the average category of exercise at each time point, the IPAQ was administered was “moderate”. Stress, measured by the PSS, was not significantly different between intervention periods (*p* = 0.61, Table 2). No adverse events could be attributed to the probiotic intervention. Three participants experienced feelings of nausea from the aspirin challenge. All participants were considered compliant. Of the participants who consumed the probiotic first, 67% believed they had the probiotic first and 33% believed they had the placebo first. For those who consumed the placebo first, 50% believed they had the probiotic first and 50% believed they had the placebo first (*p* = 0.46). This study was properly blinded.

**Table 1:** Participant demographics and compliance

	*n* = 29
Sex, n (%)	
Male	13 (45)
Female	16 (55)
Age, y	26.0 ± 5.5^a^
Race, n (%)	
Asian	5 (18)
Black/African American	1 (3)
Other	3 (10)
White	20 (69)
BMI^b^, kg/m^2^	24.1 ± 3.3
Days questionnaires completed^c^, %	98 ± 0.01
Days of correct supplement intake^c^, %	98 ± 0.01

^a^Mean ± SD (all such values)

^b^BMI, Body Mass Index

^c^Participants were considered compliant if they completed at least 95% of their daily and weekly questionnaires and reported consumption of at least 90% of their required study supplements

**Table 2:** Perceived stress, dietary fiber intake, and physical activity during the 3-week intervention period

	Placebo (*n* = 29)	Probiotic (*n* = 29)	*P* Value^1^
PSS^a^	19.5 ± 0.6^b^	20 ± 0.4	0.61
Dietary fiber intake (g)^c^	18.7 ± 0.5	18.6 ± 0.5	0.50
IPAQ (MET minutes)^d^	2912 ± 284	2874 ± 439	0.29

^1^Variables were analyzed by using a paired t-test

^a^Perceived Stress Scale (PSS) is the sum of a 10-item questionnaire that assesses the frequency of stressful events over the previous month on a scale of never (0) to very often (4), for each of the 10 questions for a total score of 40 The PSS was administered at visits 4 and 8 to assess stress over each intervention period

^b^Mean ± SEM

^c^Dietary fiber intake over the past week was estimated using the Block Fiber Screener [47]. The fiber screener was administered each week during each intervention. Scores were averaged across all 3 weeks for each intervention

^d^The International Physical Activity Question (IPAQ) asks participants to report the time over the previous week that they engaged in various activities. Data were transcribed to metabolic equivalents (MET) in minutes. The IPAQ was administered each week during each intervention. Scores were averaged across all 3 weeks for each intervention

### Intestinal permeability

**Fig. 2:**
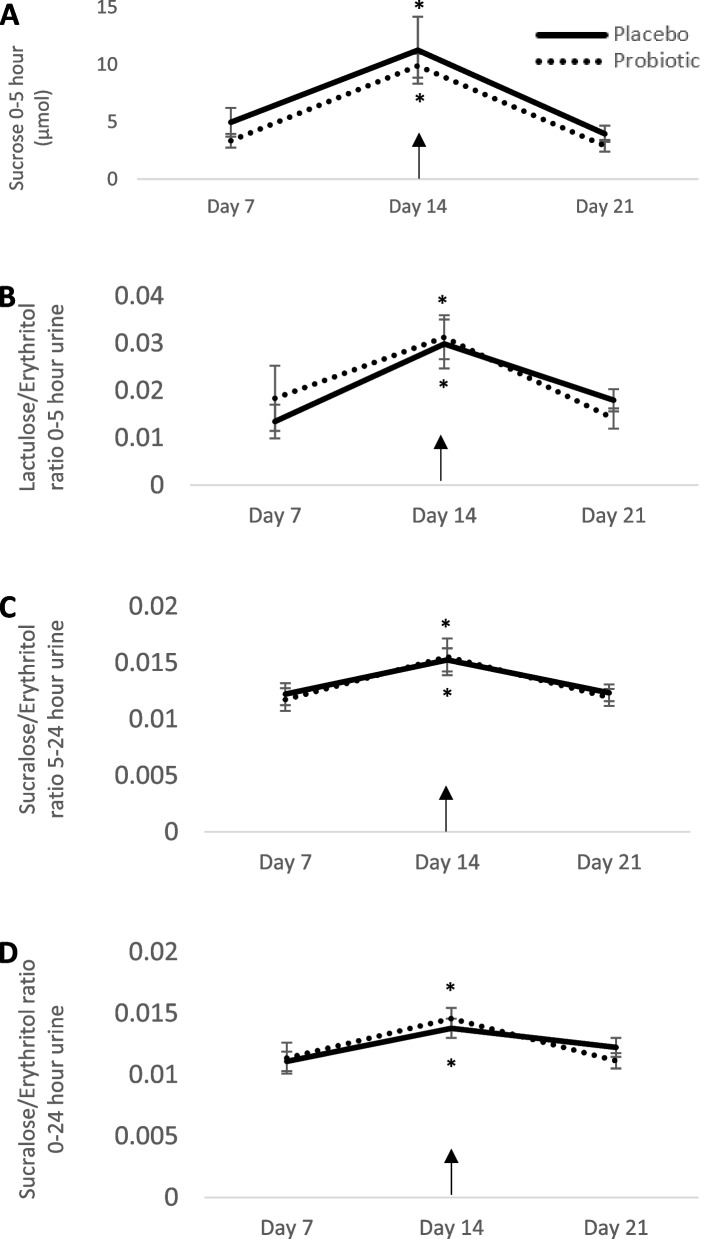
Gastroduodenal permeability, small intestinal permeability, colonic permeability, and whole gut permeability are reported (*n* = 29). Gastroduodenal permeability (**A**) is assessed by measuring the change in sucrose recovery from various time points while small intestinal (**B**), colonic (**C**), and whole gut permeability (**D**) are assessed by ratios of sugar recovery from various time points. The probiotic is represented by the dashed line. The placebo is represented by the solid line. Day 7 and Day 21 represent 7 and 21 days on the probiotic or placebo, respectively. These days do not include an aspirin challenge. Day 14, indicated by the arrow, represents the aspirin challenge. Each participant is represented twice in each figure (i.e., probiotic and placebo arms). *p < 0.05 vs all other time points

The change in gastroduodenal permeability between the aspirin challenge and the previous week without an aspirin challenge was not significant between the probiotic (6.5 ± 1.2 μmol) and placebo (6.3 ± 3.2 μmol; *p* = 0.92). Additionally, the order by intervention effect was not significant suggesting that there was no carryover effect with the crossover. Sucrose excretion significantly increased following aspirin challenge (Fig. 2A) with no differences between interventions. Small intestinal permeability (*p* = 0.62, Fig. 2B), colonic permeability (0.72, Fig. 2C), and whole gut permeability (*p* = 0.79, Fig. 2D) were not different between the probiotic and placebo (Supplemental Table 1). There was, however, a significant effect of week for small intestinal (*p* < 0.0001), colonic (*p* < 0.0001), and whole gut (*p* < 0.0001) permeability, indicating that intestinal permeability significantly increased with the dose of aspirin that was provided to the participants.

### Questionnaire data

When analyzing the weekly GSRS syndrome and total scores at week 1, 2, and 3 of each intervention, there were no significant differences across weeks or between the probiotic and placebo (Table 3). The DQLQ scores overall were significantly lower across all weeks on the probiotic (0.45 ± 0.04) compared to the placebo (0.68 ± 0.05; *p* < 0.05), indicating a better quality of life score. There were no significant effects of the probiotic intervention for any outcomes recorded on the daily questionnaire including bowel frequency, BSFS, and daily stress (Table 3).

**Table 3:** Daily and weekly questionnaire responses and corresponding *P* values of effects included in the analyses^1^

	**Baseline**	**Week 1**	**Week 2**	**Week 3**	***P* Value**^2^
Total GSRS^a^					W: 0.33
Placebo	23.2 ± 1.28	22.5 ± 1.42	22.9 ± 1.46	22.4 ± 1.52	I: 0.56
Probiotic	21.2 ± 1.36	21.7 ± 1.30	21.7 ± 1.35	20.9 ± 1.18	WxI: 0.74
Abdominal Pain^b^					W: 0.56
Placebo	1.50 ± 0.08	1.48 ± 0.13	1.49 ± 0.10	1.48 ± 0.11	I: 0.39
Probiotic	1.37 ± 0.08	1.36 ± 0.10	1.49 ± 0.13	1.38 ± 0.09	WxI: 0.66
Diarrhea^c^					W: 0.58
Placebo	1.43 ± 0.11	1.40 ± 0.12	1.38 ± 0.09	1.37 ± 0.12	I: 0.53
Probiotic	1.37 ± 0.10	1.28 ± 0.09	1.36 ± 0.09	1.25 ± 0.10	WxI: 0.59
Constipation^d^					W: 0.75
Placebo	1.61 ± 0.11	1.50 ± 0.11	1.58 ± 0.15	1.58 ± 0.13	I: 0.97
Probiotic	1.39 ± 0.09	1.47 ± 0.11	1.45 ± 0.11	1.34 ± 0.08	WxI: 0.48
Indigestion^e^					W: 0.28
Placebo	1.84 ± 0.16	1.79 ± 0.16	1.79 ± 0.16	1.73 ± 0.16	I: 0.43
Probiotic	1.64 ± 0.16	1.78 ± 0.16	1.63 ± 0.15	1.67 ± 0.15	WxI: 0.39
Reflux^f^					W: 0.52
Placebo	1.13 ± 0.05	1.08 ± 0.04	1.22 ± 0.08	1.12 ± 0.05	I: 0.73
Probiotic	1.15 ± 0.05	1.14 ± 0.05	1.12 ± 0.04	1.16 ± 0.07	WxI: 0.28
DQLQ^g^					W: 0.06
Placebo	6.9 ± 2.0	7.8 ± 2.5	7.7 ± 1.9	5.9 ± 2.1	I: 0.01
Probiotic	6.3 ± 2.2	4.6 ± 1.8	5.4 ± 2.3	4.1 ± 1.6	WxI: 0.62
BM^h^					W: 0.87
Placebo	1.2 ± 0.1	1.2 ± 0.1	1.2 ± 0.1	1.1 ± 0.1	I: 0.94
Probiotic	1.2 ± 0.1	1.2 ± 0.1	1.2 ± 0.1	1.2 ± 0.1	WxI: 0.37
BSFS^i^					W: 0.94
Placebo	3.6 ± 0.2	3.7 ± 0.2	3.6 ± 0.2	3.6 ± 0.2	I: 0.34
Probiotic	3.5 ± 0.1	3.4 ± 0.1	3.5 ± 0.2	3.5 ± 0.2	WxI: 0.67
Stress^j^					W: 0.29
Placebo	3.3 ± 0.3	3.6 ± 0.3	3.6 ± 0.3	3.8 ± 0.4	I: 0.88
Probiotic	3.3 ± 0.3	3.5 ± 0.3	3.6 ± 0.4	3.8 ± 0.4	WxI: 0.73

^1^Data represent gastrointestinal symptoms, digestion-associated quality of life, bowel movement frequency, stool form, and stress by week for each intervention. There were 3 weeks in each intervention. Baseline represents the mean score during the 2 weeks leading up to each intervention. Data were analyzed in a general linear mixed model including a random effect of participant to account for repeated measures. Baseline values were included in the model to control for potential differences in individuals at baseline. Sex was included in the model, but no interactions between sex and intervention were observed. Regardless of significance, all main effects remained in the model

^2^W, week; I, intervention; WxI, week by intervention

^a^The GSRS is a 15-item questionnaire that assesses gastrointestinal symptoms on a scale from 1 (no discomfort at all) to 7 (very severe discomfort) for five syndromes which include abdominal pain, diarrhea, constipation, indigestion, and reflux. The total represents the sum of all 15 symptoms

^b^Abdominal pain score represents the mean score from abdominal pain, hunger pains, and nausea

^c^Diarrhea syndrome score represents the mean score from diarrhea, loose stools, and urgent need for defecation

^d^Constipation syndrome score represents the mean score from constipation, hard stools, and feeling of incomplete evacuation

^e^Indigestion syndrome score represents the mean score from rumbling, bloating, burping, and gas

^f^Reflux syndrome score represents the mean score from heartburn and acid regurgitation

^g^The Digestion-Associated Quality of Life Questionnaire is comprised of nine questions that assess digestive events and experiences and is scored from never (zero- 0% of the time) to always (1- 100% of the time)

^h^BM represents the mean number of bowel movements each week

^i^BSFS is the mean daily Bristol Stool Form Scale is a measure of stool consistency (1- hard stool, 7- watery stool)

^j^Mean daily stress (0- no stress, 10- extremely stressed)

### Microbiota species of interest

The mean fold change in abundance of microbes of interest across the probiotic intervention with respect to the placebo for *F. prausnitzii* trended towards significance with higher abundance when participants (*n* = 29) consumed the probiotic (1.90 ± 0.40; *p* = 0.07). The mean fold changes in quantification of *A. muciniphila* (0.88 ± 0.12; *p* = 0.28), *Roseburia* spp. (1.55 ± 0.31; *p* = 0.26), and the *Bifidobacterium* spp. (1.03 ± 0.14; *p *= 0.75) were not significantly different from 1 (i.e., no change) for the probiotic with respect to the placebo intervention.

## Discussion

The purpose of this study was to determine whether prophylactic probiotic supplementation could mitigate the GI permeability induced by an acute aspirin challenge. Gastroduodenal, small intestinal, colonic, and whole gut permeability increased with aspirin challenge, but prophylactic supplementation of the probiotic combination did not impact this change. It was hypothesized that the *Lactobacillus* species contained within the supplement could attenuate aspirin-induced mucosal damage by impacting mucosal immunity and maintaining tight junction integrity. Previous preclinical studies have demonstrated that these specific probiotic strains have protective effects on the gut barrier. In vitro, this probiotic adheres to intestinal epithelial cells thereby decreasing pathogen adhesion [31]. These strains also prevent the loss of trans-epithelial electrical resistance with a pathogen challenge and are suggested to play a role in barrier defense by maintaining the intestinal mucus layer [31, 57–59]. Both strains downregulate pro-inflammatory cytokines in vitro [29, 32, 57]. In vivo, bacterial translocation to the mesenteric lymph nodes in a rat stress model was prevented by pretreatment with this probiotic combination [60].

Although this probiotic may provide potential benefits in maintaining GI integrity, it was not effective in this particular model utilizing an aspirin challenge. It is possible that the effect of *Lactobacilli* on barrier function is strain-specific and importantly, challenge-specific [61]. For example, Luyer and colleagues showed that *L. rhamnosus* and *L. fermentum* similarly inhibited adherence of pathogens to epithelial cells and endotoxin-induced inflammation in vitro, but only *L. rhamnosus* reduced loss of gut barrier function in rats prophylactically treated with the probiotics prior to inducing hemorrhagic shock [61]. Additionally, the selected aspirin dose (975 mg 12 h the night before, with fasting, and 975 mg 30 min before assessing GI permeability) could have been too high for the probiotic to significantly reduce damage. However, Krumbeck and colleagues administered 1300 mg of aspirin 12 and 24 h before assessing GI barrier function and observed a reduction in intestinal permeability with 3 weeks of prophylactic supplementation with either *Bifidobacterium adolescentis* IVS-1 or *B. lactis* BB-12 [62]. In the current study, we supplemented participants with the probiotic combination for 2 weeks before administering the aspirin challenge. It is possible that a longer intervention or a higher dose of this probiotic is required to impact aspirin-induced increases in GI permeability.

This study examined four bacterial species of interest in the weekly stool samples across each intervention. *F. prausnitzii*, *A. muciniphila*, *Roseburia* spp., and *Bifidobacterium* spp. were selected as species of interests as these microbes may maintain intestinal permeability by increasing butyrate and mucin production, preventing inflammation, and normalizing tight junction integrity [63–66]. During the 3-week interventions, the fold change of *A. muciniphila*, *Roseburia* spp., and *Bifidobacterium* spp. was not different for the probiotic with respect to the placebo, however, a trend was observed in of *F. prausnitzii*. *F. prausnitzii* is considered a next-generation species due to its ability to potentially prevent and treat various diseases [67]. *F. prausnitzii* is a prominent butyrate producer, providing energy for intestinal cells and decreasing inflammation [64, 68]. Future work should investigate if *F. prausnitzii* is one of the contributing factors by which this probiotic has exhibited its beneficial effects on the gut barrier in preclinical models.

A novel finding in the current study was that digestion-associated quality of life was better (i.e., statistically significant) when participants were taking the probiotic combination versus the placebo. However, the biological significance of this has yet to be determined because GI symptoms were not different between interventions.

Strengths of this study include that participants were compliant; therefore, data were analyzed on an intent-to-treat basis. Additionally, there was a 4-week washout period between arms of the crossover, which appears to be sufficient to prevent a carryover effect [69]. This study also has limitations. Unfortunately, it was discovered during the post-study analyses of the urine samples that due to a manufacturer packaging error, neotame was administered in the 5-sugar cocktail in place of L-rhamnose. Considering this, erythritol was used instead of L-rhamnose in accordance with previously described methods [50]. Other studies have used erythritol in place of L-rhamnose to measure small intestinal permeability as they both have similar molecular weight (erythritol 122.1 g/mol vs L-rhamnose 164.2 g/mol), both are absorbed readily in the proximal small intestine by diffusion through porins, and neither are fermented by the human microbiota [70].

While previous preclinical studies using a number of models show that this probiotic can maintain gut barrier functions, this dose of the probiotic combination was ineffective in preventing or mitigating the effects on GI permeability in this model using generally healthy participants and an aspirin challenge. Digestion-associated quality of life was significantly better when consuming the probiotic, which deserves further investigation as does the effect of the probiotic on fecal *F. prausnitzii*.

### Supplementary Information

**Additional file 1: Supplemental Table 1. **Urinary output of sugar probes 5 and 24 hours after consuming the sugar cocktail.

## Data Availability

The datasets used and/or analyzed during the current study are available from the corresponding author upon reasonable request and approval of the University of Florida.

## Abbreviations

BSFS: Bristol stool form scale
DQLQ: Digestion-Associated Quality of Life Questionnaire
GI: Gastrointestinal
GSRS: Gastrointestinal Symptom Rating Scale
NSAID: Nonsteroidal Anti-Inflammatory Drug
qPCR: Quantitative Polymerase Chain Reaction
