# Supplementary material for: The effect of an acute aspirin challenge on intestinal permeability in healthy adults with and without prophylactic probiotic consumption: a double-blind, placebo-controlled, randomized trial
Source: BMC Gastroenterol. 2024 Jan 2;24:4. doi: 10.1186/s12876-023-03102-w (PMC10759586; doi:10.1186/s12876-023-03102-w)
Supplement: Supplementary file 1 — Additional file 1: Supplemental Table 1. Urinary output of sugar probes 5 and 24 hours after consuming the sugar cocktail. [file 12876_2023_3102_MOESM1_ESM.docx]

**Supplemental Table 1.** Urinary output of sugar probes 5 and 24 hours after consuming the sugar cocktail^1^


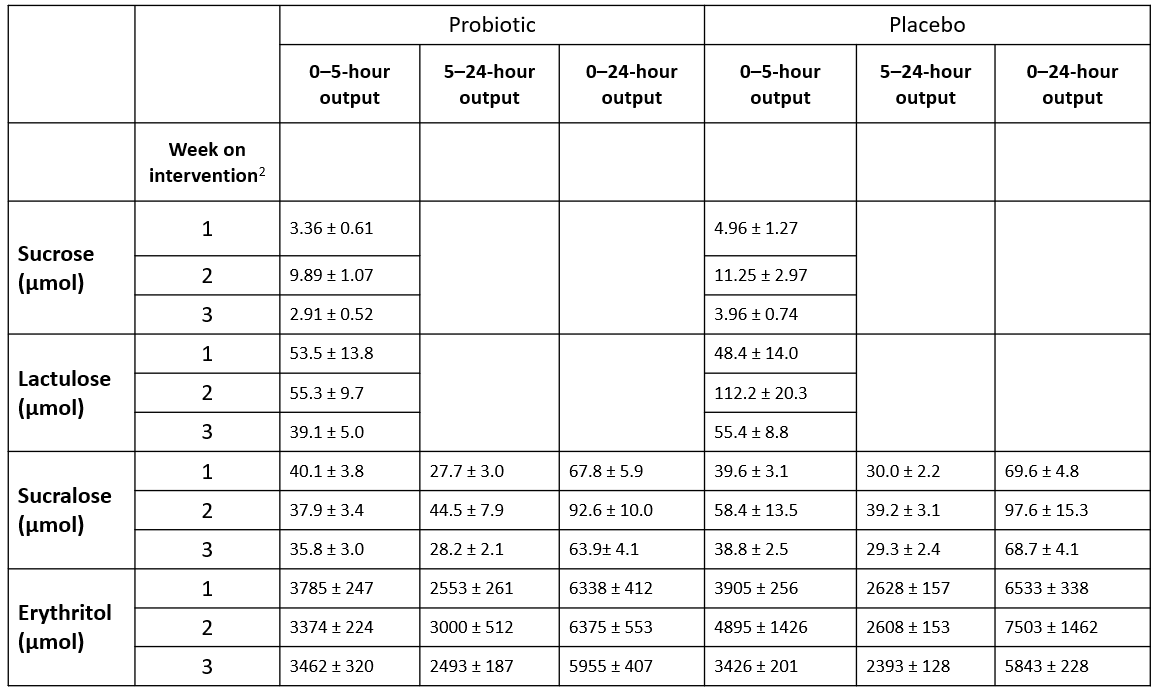


^1^An intestinal permeability test was conducted using a sugar cocktail on days 7, 14, and 21 after beginning the study capsules during each intervention period. The sugar cocktail consisted of 150 mL of water with 1 g of sucrose, 1 g of lactulose, 1 g of sucralose, and 1 g of erythritol. Participants were asked to fast at least 8 hours prior to their visit time. Prior to each permeability test, participants were asked to empty their bladder and then drink the sugar cocktail. Urine was collected for the first 5 hours while the participant remained fasted. Separate urine collection jars were used for the first 5 hours (0-5-hour) and the remaining 19 hours (5-24-hour) of the 24-hour (0-24-hour) collection. Sugar probes in the urine were quantitated using liquid chromatography with tandem mass spectrometry.

^2^Permeabiltiy testing was completed on days 7 (week 1 on the intervention), 14 (week 2 on the intervention), and 21 (week 3 on the intervention). Day 14 (week 2 on the intervention) included an aspirin challenge. Participants were asked to consume 975 mg of aspirin before bed the night before the test. Thirty minutes before beginning the day 14 intestinal permeability test and while still fasting, participants consumed an additional 975 mg of aspirin.
